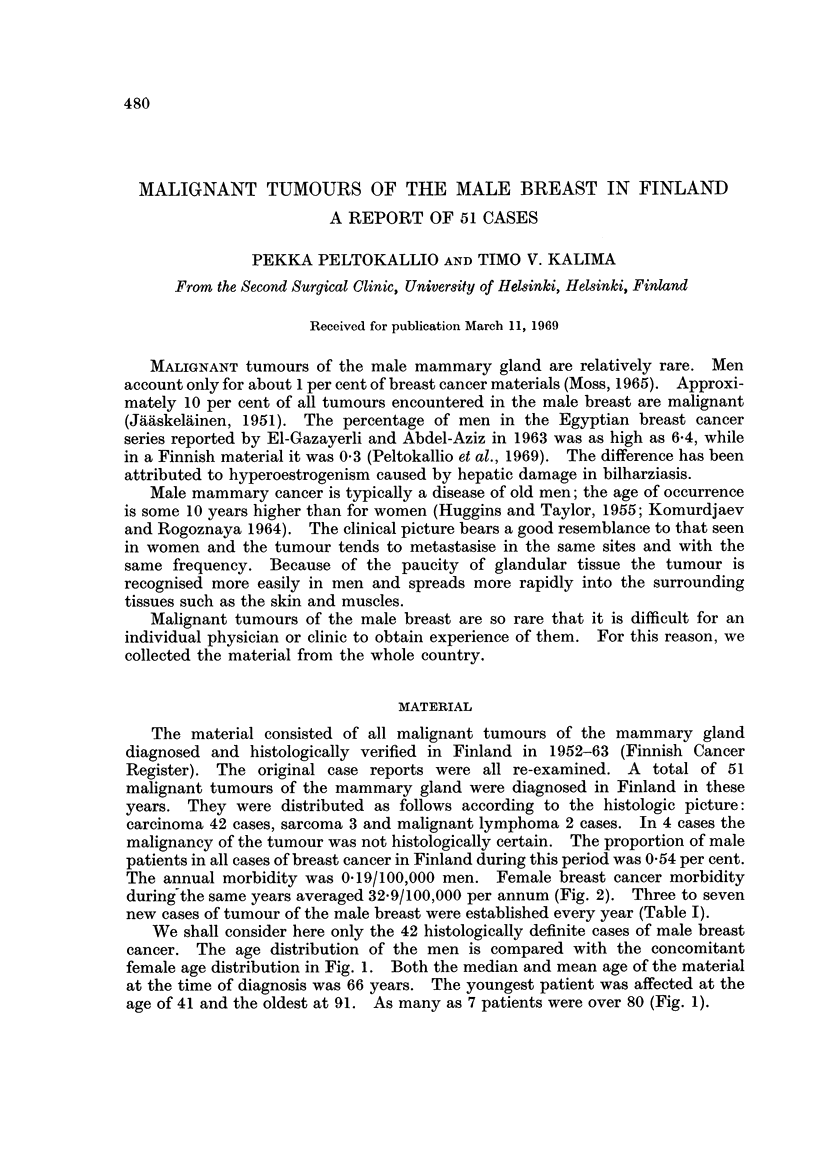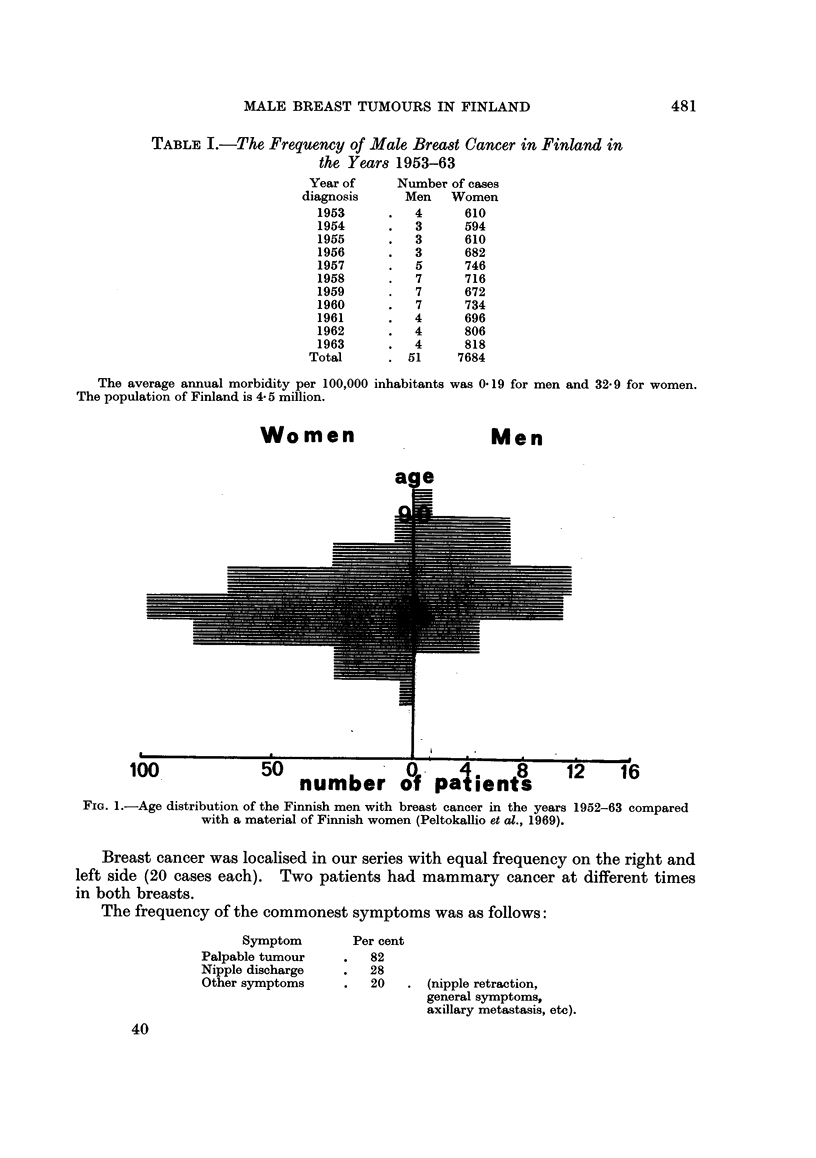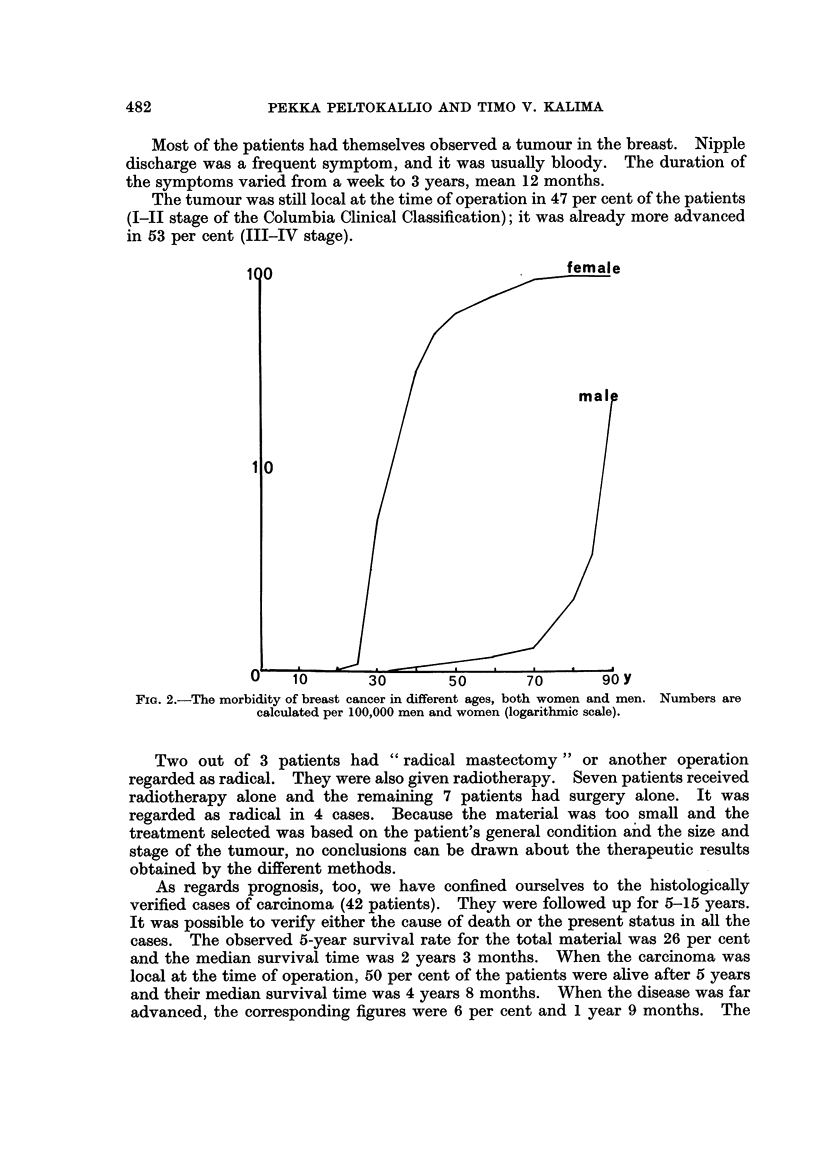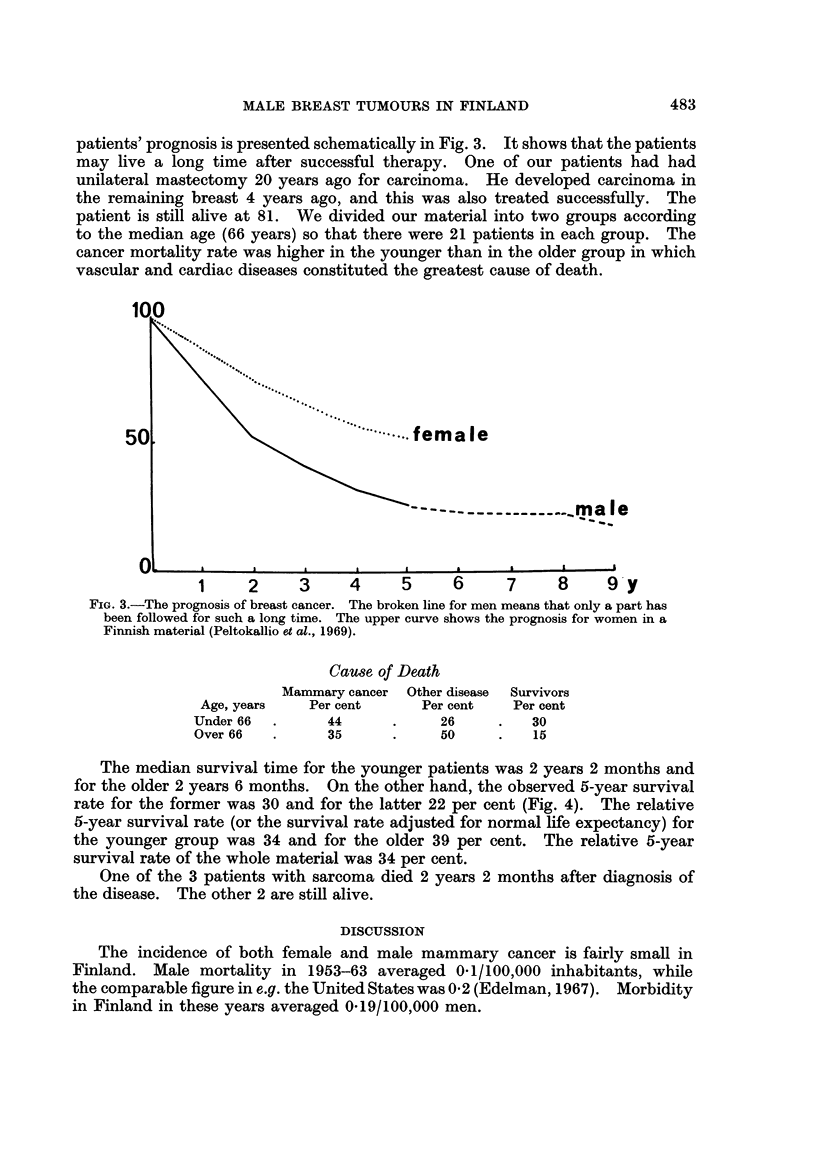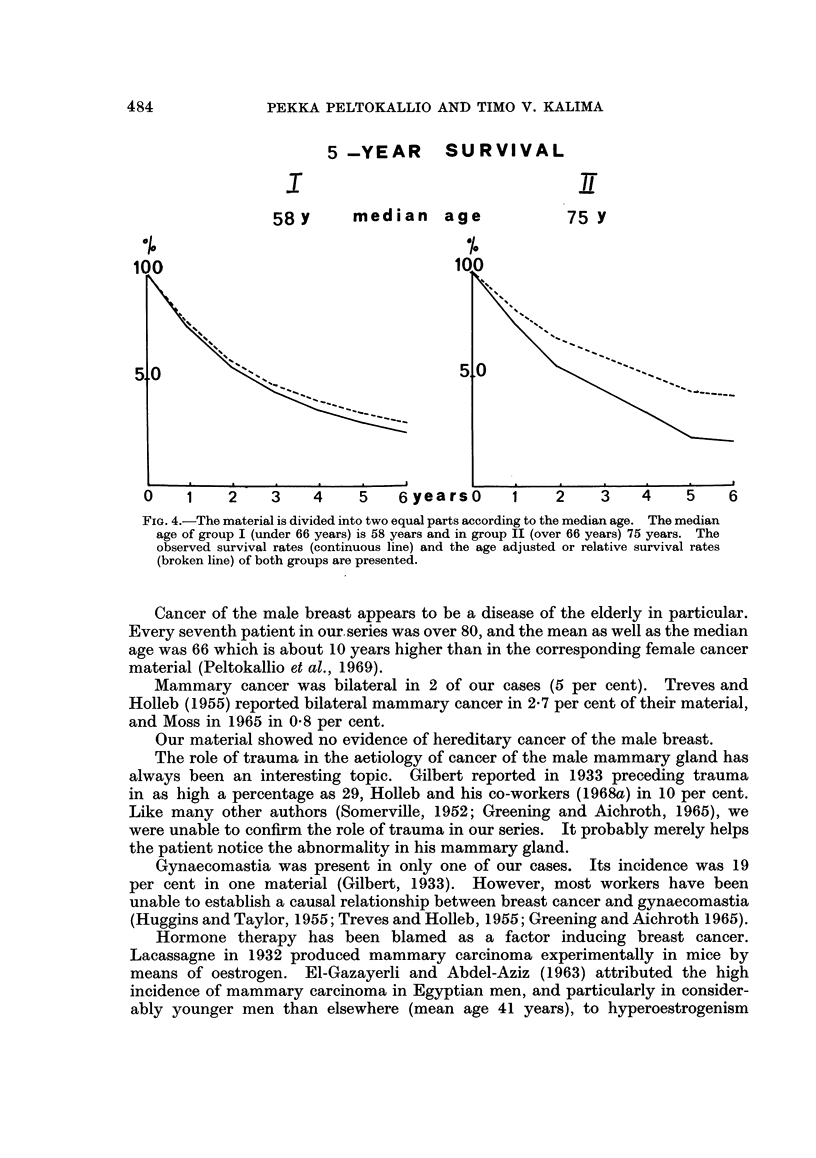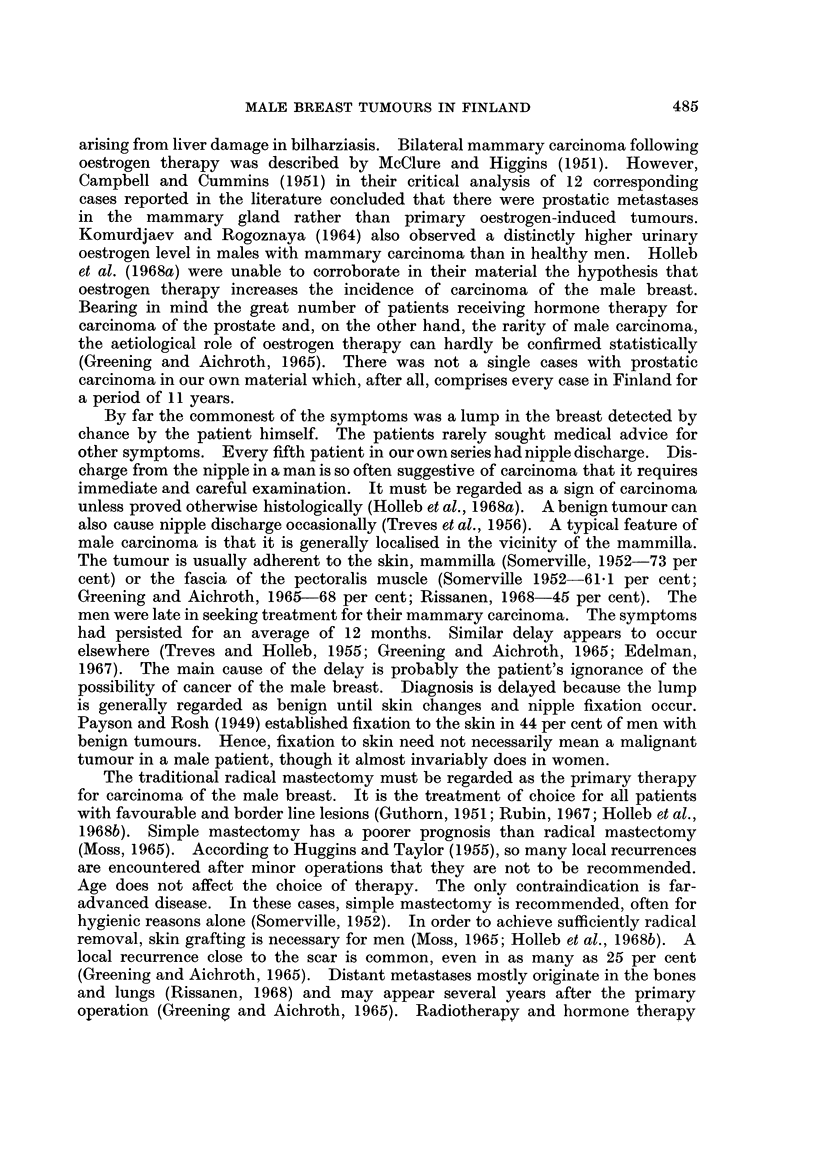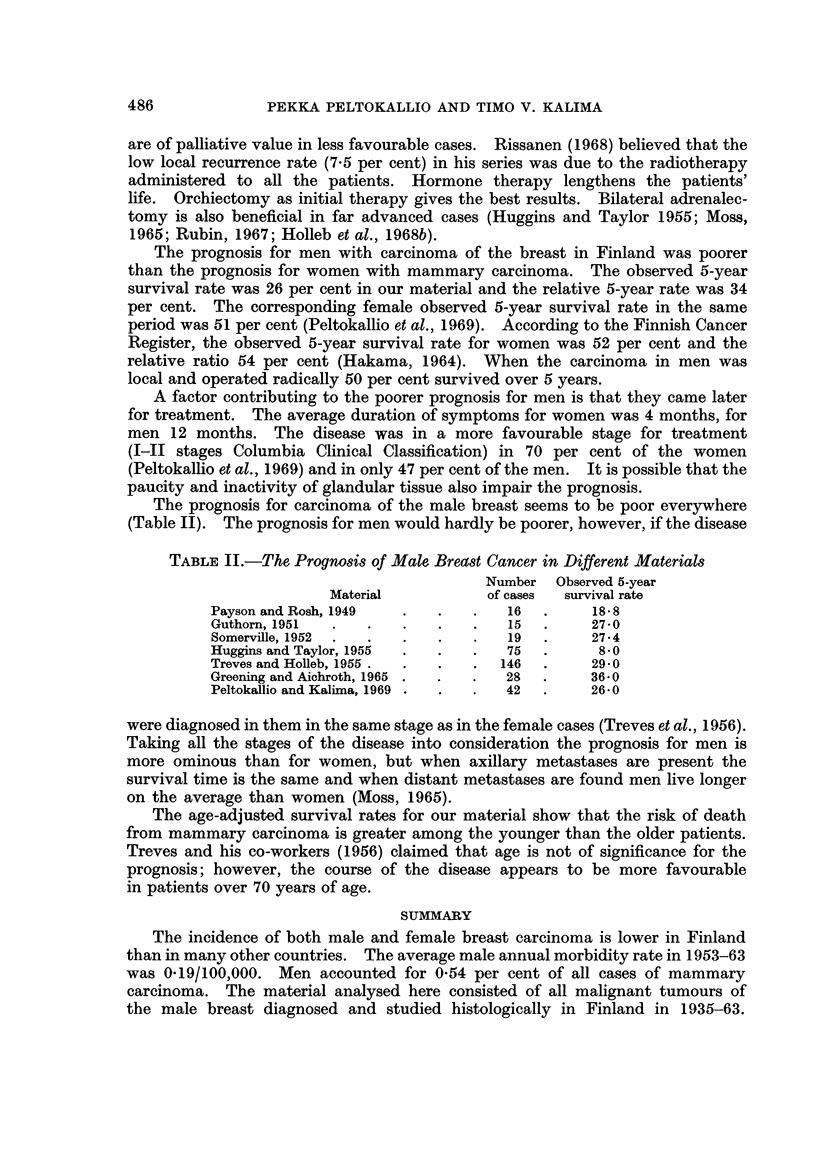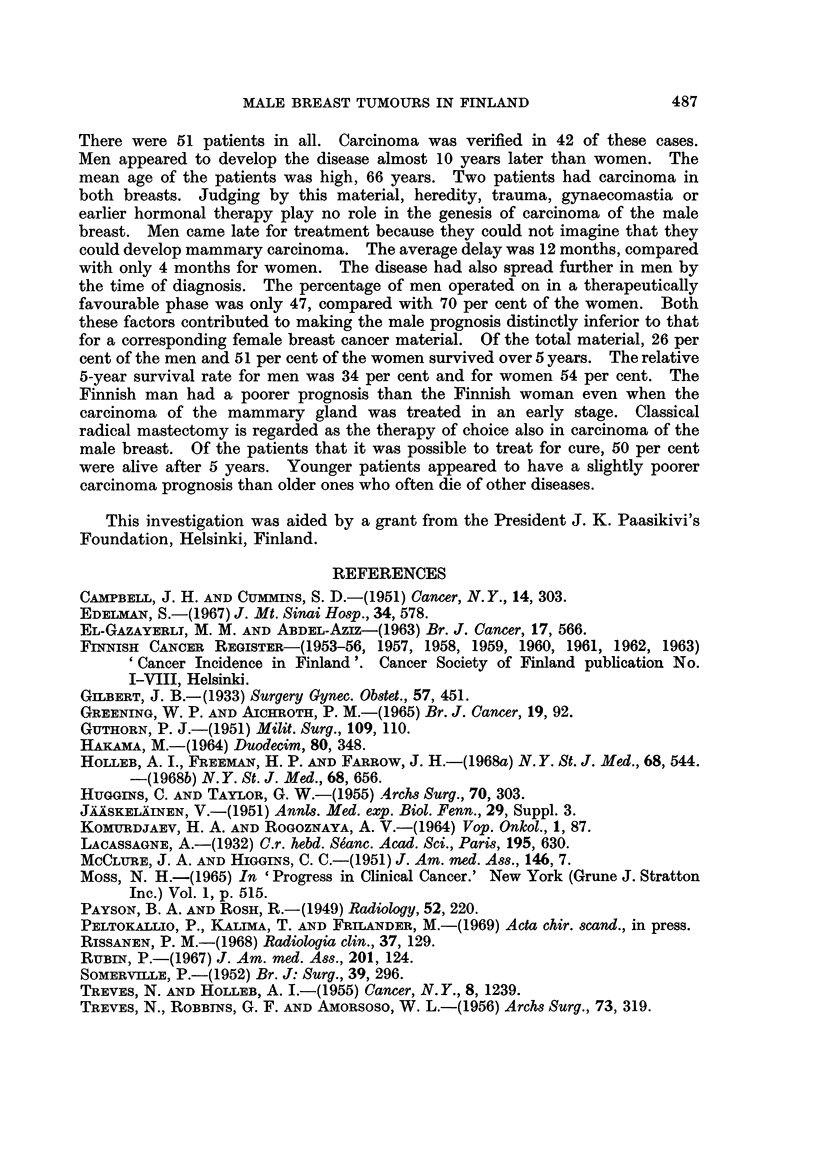# Malignant tumours of the male breast in Finland. A report of 51 cases.

**DOI:** 10.1038/bjc.1969.60

**Published:** 1969-09

**Authors:** P. Peltokallio, T. V. Kalima


### MALIGNANT TUMOURS OF THE MALE BREAST IN FINLAND
### A REPORT OF 51 CASES

PEKKA PELTOKALLIO AND TIMO V. KALIMA
*From the Second Surgical Clinic, University of Helsinki, Helsinki, Finland*

Received for publication March 11, 1969

MALIGNANT tumours of the male mammary gland are relatively rare. Men account only for about 1 per cent of breast cancer materials (Moss, 1965). Approximately 10 per cent of all tumours encountered in the male breast are malignant (Jääskeläinen, 1951). The percentage of men in the Egyptian breast cancer series reported by El-Gazayerli and Abdel-Aziz in 1963 was as high as 6·4, while in a Finnish material it was 0·3 (Peltokallio *et al.*, 1969). The difference has been attributed to hyperoestrogenism caused by hepatic damage in bilharziasis.

Male mammary cancer is typically a disease of old men; the age of occurrence is some 10 years higher than for women (Huggins and Taylor, 1955; Komurdjaev and Rogoznaya 1964). The clinical picture bears a good resemblance to that seen in women and the tumour tends to metastasise in the same sites and with the same frequency. Because of the paucity of glandular tissue the tumour is recognised more easily in men and spreads more rapidly into the surrounding tissues such as the skin and muscles.

Malignant tumours of the male breast are so rare that it is difficult for an individual physician or clinic to obtain experience of them. For this reason, we collected the material from the whole country.

### MATERIAL

The material consisted of all malignant tumours of the mammary gland diagnosed and histologically verified in Finland in 1952–63 (Finnish Cancer Register). The original case reports were all re-examined. A total of 51 malignant tumours of the mammary gland were diagnosed in Finland in these years. They were distributed as follows according to the histologic picture: carcinoma 42 cases, sarcoma 3 and malignant lymphoma 2 cases. In 4 cases the malignancy of the tumour was not histologically certain. The proportion of male patients in all cases of breast cancer in Finland during this period was 0·54 per cent. The annual morbidity was 0·19/100,000 men. Female breast cancer morbidity during the same years averaged 32·9/100,000 per annum (Fig. 2). Three to seven new cases of tumour of the male breast were established every year (Table I).

We shall consider here only the 42 histologically definite cases of male breast cancer. The age distribution of the men is compared with the concomitant female age distribution in Fig. 1. Both the median and mean age of the material at the time of diagnosis was 66 years. The youngest patient was affected at the age of 41 and the oldest at 91. As many as 7 patients were over 80 (Fig. 1).

TABLE I.—*The Frequency of Male Breast Cancer in Finland in the Years* 1953–63

| Year of diagnosis | Number of cases Men | Women |
|---|---|---|
| 1953 | 4 | 610 |
| 1954 | 3 | 594 |
| 1955 | 3 | 610 |
| 1956 | 3 | 682 |
| 1957 | 5 | 746 |
| 1958 | 7 | 716 |
| 1959 | 7 | 672 |
| 1960 | 7 | 734 |
| 1961 | 4 | 696 |
| 1962 | 4 | 806 |
| 1963 | 4 | 818 |
| Total | 51 | 7684 |

The average annual morbidity per 100,000 inhabitants was 0·19 for men and 32·9 for women. The population of Finland is 4·5 million.

FIG. 1.—Age distribution of the Finnish men with breast cancer in the years 1952–63 compared with a material of Finnish women (Peltokallio *et al.*, 1969).

Breast cancer was localised in our series with equal frequency on the right and left side (20 cases each). Two patients had mammary cancer at different times in both breasts.

The frequency of the commonest symptoms was as follows:

| Symptom | Per cent | |
|---|---|---|
| Palpable tumour | 82 | |
| Nipple discharge | 28 | |
| Other symptoms | 20 | (nipple retraction, general symptoms, axillary metastasis, etc). |

Most of the patients had themselves observed a tumour in the breast. Nipple discharge was a frequent symptom, and it was usually bloody. The duration of the symptoms varied from a week to 3 years, mean 12 months.

The tumour was still local at the time of operation in 47 per cent of the patients (I–II stage of the Columbia Clinical Classification); it was already more advanced in 53 per cent (III–IV stage).

Fig. 2.—The morbidity of breast cancer in different ages, both women and men. Numbers are calculated per 100,000 men and women (logarithmic scale).

Two out of 3 patients had "radical mastectomy" or another operation regarded as radical. They were also given radiotherapy. Seven patients received radiotherapy alone and the remaining 7 patients had surgery alone. It was regarded as radical in 4 cases. Because the material was too small and the treatment selected was based on the patient's general condition and the size and stage of the tumour, no conclusions can be drawn about the therapeutic results obtained by the different methods.

As regards prognosis, too, we have confined ourselves to the histologically verified cases of carcinoma (42 patients). They were followed up for 5–15 years. It was possible to verify either the cause of death or the present status in all the cases. The observed 5-year survival rate for the total material was 26 per cent and the median survival time was 2 years 3 months. When the carcinoma was local at the time of operation, 50 per cent of the patients were alive after 5 years and their median survival time was 4 years 8 months. When the disease was far advanced, the corresponding figures were 6 per cent and 1 year 9 months. The patients' prognosis is presented schematically in Fig. 3. It shows that the patients may live a long time after successful therapy. One of our patients had had unilateral mastectomy 20 years ago for carcinoma. He developed carcinoma in the remaining breast 4 years ago, and this was also treated successfully. The patient is still alive at 81. We divided our material into two groups according to the median age (66 years) so that there were 21 patients in each group. The cancer mortality rate was higher in the younger than in the older group in which vascular and cardiac diseases constituted the greatest cause of death.

FIG. 3.—The prognosis of breast cancer. The broken line for men means that only a part has been followed for such a long time. The upper curve shows the prognosis for women in a Finnish material (Peltokallio *et al.*, 1969).

*Cause of Death*

| Age, years | Mammary cancer Per cent | Other disease Per cent | Survivors Per cent |
|---|---|---|---|
| Under 66 | 44 | 26 | 30 |
| Over 66 | 35 | 50 | 15 |

The median survival time for the younger patients was 2 years 2 months and for the older 2 years 6 months. On the other hand, the observed 5-year survival rate for the former was 30 and for the latter 22 per cent (Fig. 4). The relative 5-year survival rate (or the survival rate adjusted for normal life expectancy) for the younger group was 34 and for the older 39 per cent. The relative 5-year survival rate of the whole material was 34 per cent.

One of the 3 patients with sarcoma died 2 years 2 months after diagnosis of the disease. The other 2 are still alive.

### DISCUSSION

The incidence of both female and male mammary cancer is fairly small in Finland. Male mortality in 1953–63 averaged 0·1/100,000 inhabitants, while the comparable figure in *e.g.* the United States was 0·2 (Edelman, 1967). Morbidity in Finland in these years averaged 0·19/100,000 men.

FIG. 4.—The material is divided into two equal parts according to the median age. The median age of group I (under 66 years) is 58 years and in group II (over 66 years) 75 years. The observed survival rates (continuous line) and the age adjusted or relative survival rates (broken line) of both groups are presented.

Cancer of the male breast appears to be a disease of the elderly in particular. Every seventh patient in our series was over 80, and the mean as well as the median age was 66 which is about 10 years higher than in the corresponding female cancer material (Peltokallio *et al.*, 1969).

Mammary cancer was bilateral in 2 of our cases (5 per cent). Treves and Holleb (1955) reported bilateral mammary cancer in 2·7 per cent of their material, and Moss in 1965 in 0·8 per cent.

Our material showed no evidence of hereditary cancer of the male breast.

The role of trauma in the aetiology of cancer of the male mammary gland has always been an interesting topic. Gilbert reported in 1933 preceding trauma in as high a percentage as 29, Holleb and his co-workers (1968*a*) in 10 per cent. Like many other authors (Somerville, 1952; Greening and Aichroth, 1965), we were unable to confirm the role of trauma in our series. It probably merely helps the patient notice the abnormality in his mammary gland.

Gynaecomastia was present in only one of our cases. Its incidence was 19 per cent in one material (Gilbert, 1933). However, most workers have been unable to establish a causal relationship between breast cancer and gynaecomastia (Huggins and Taylor, 1955; Treves and Holleb, 1955; Greening and Aichroth 1965).

Hormone therapy has been blamed as a factor inducing breast cancer. Lacassagne in 1932 produced mammary carcinoma experimentally in mice by means of oestrogen. El-Gazayerli and Abdel-Aziz (1963) attributed the high incidence of mammary carcinoma in Egyptian men, and particularly in considerably younger men than elsewhere (mean age 41 years), to hyperoestrogenism arising from liver damage in bilharziasis. Bilateral mammary carcinoma following oestrogen therapy was described by McClure and Higgins (1951). However, Campbell and Cummins (1951) in their critical analysis of 12 corresponding cases reported in the literature concluded that there were prostatic metastases in the mammary gland rather than primary oestrogen-induced tumours. Komurdjaev and Rogoznaya (1964) also observed a distinctly higher urinary oestrogen level in males with mammary carcinoma than in healthy men. Holleb *et al.* (1968*a*) were unable to corroborate in their material the hypothesis that oestrogen therapy increases the incidence of carcinoma of the male breast. Bearing in mind the great number of patients receiving hormone therapy for carcinoma of the prostate and, on the other hand, the rarity of male carcinoma, the aetiological role of oestrogen therapy can hardly be confirmed statistically (Greening and Aichroth, 1965). There was not a single cases with prostatic carcinoma in our own material which, after all, comprises every case in Finland for a period of 11 years.

By far the commonest of the symptoms was a lump in the breast detected by chance by the patient himself. The patients rarely sought medical advice for other symptoms. Every fifth patient in our own series had nipple discharge. Discharge from the nipple in a man is so often suggestive of carcinoma that it requires immediate and careful examination. It must be regarded as a sign of carcinoma unless proved otherwise histologically (Holleb *et al.*, 1968*a*). A benign tumour can also cause nipple discharge occasionally (Treves *et al.*, 1956). A typical feature of male carcinoma is that it is generally localised in the vicinity of the mammilla. The tumour is usually adherent to the skin, mammilla (Somerville, 1952—73 per cent) or the fascia of the pectoralis muscle (Somerville 1952—61·1 per cent; Greening and Aichroth, 1965—68 per cent; Rissanen, 1968—45 per cent). The men were late in seeking treatment for their mammary carcinoma. The symptoms had persisted for an average of 12 months. Similar delay appears to occur elsewhere (Treves and Holleb, 1955; Greening and Aichroth, 1965; Edelman, 1967). The main cause of the delay is probably the patient's ignorance of the possibility of cancer of the male breast. Diagnosis is delayed because the lump is generally regarded as benign until skin changes and nipple fixation occur. Payson and Rosh (1949) established fixation to the skin in 44 per cent of men with benign tumours. Hence, fixation to skin need not necessarily mean a malignant tumour in a male patient, though it almost invariably does in women.

The traditional radical mastectomy must be regarded as the primary therapy for carcinoma of the male breast. It is the treatment of choice for all patients with favourable and border line lesions (Guthorn, 1951; Rubin, 1967; Holleb *et al.*, 1968*b*). Simple mastectomy has a poorer prognosis than radical mastectomy (Moss, 1965). According to Huggins and Taylor (1955), so many local recurrences are encountered after minor operations that they are not to be recommended. Age does not affect the choice of therapy. The only contraindication is far-advanced disease. In these cases, simple mastectomy is recommended, often for hygienic reasons alone (Somerville, 1952). In order to achieve sufficiently radical removal, skin grafting is necessary for men (Moss, 1965; Holleb *et al.*, 1968*b*). A local recurrence close to the scar is common, even in as many as 25 per cent (Greening and Aichroth, 1965). Distant metastases mostly originate in the bones and lungs (Rissanen, 1968) and may appear several years after the primary operation (Greening and Aichroth, 1965). Radiotherapy and hormone therapy are of palliative value in less favourable cases. Rissanen (1968) believed that the low local recurrence rate (7·5 per cent) in his series was due to the radiotherapy administered to all the patients. Hormone therapy lengthens the patients' life. Orchiectomy as initial therapy gives the best results. Bilateral adrenalectomy is also beneficial in far advanced cases (Huggins and Taylor 1955; Moss, 1965; Rubin, 1967; Holleb *et al.*, 1968*b*).

The prognosis for men with carcinoma of the breast in Finland was poorer than the prognosis for women with mammary carcinoma. The observed 5-year survival rate was 26 per cent in our material and the relative 5-year rate was 34 per cent. The corresponding female observed 5-year survival rate in the same period was 51 per cent (Peltokallio *et al.*, 1969). According to the Finnish Cancer Register, the observed 5-year survival rate for women was 52 per cent and the relative ratio 54 per cent (Hakama, 1964). When the carcinoma in men was local and operated radically 50 per cent survived over 5 years.

A factor contributing to the poorer prognosis for men is that they came later for treatment. The average duration of symptoms for women was 4 months, for men 12 months. The disease was in a more favourable stage for treatment (I–II stages Columbia Clinical Classification) in 70 per cent of the women (Peltokallio *et al.*, 1969) and in only 47 per cent of the men. It is possible that the paucity and inactivity of glandular tissue also impair the prognosis.

The prognosis for carcinoma of the male breast seems to be poor everywhere (Table II). The prognosis for men would hardly be poorer, however, if the disease

TABLE II.—*The Prognosis of Male Breast Cancer in Different Materials*

| Material | Number of cases | Observed 5-year survival rate |
|---|---|---|
| Payson and Rosh, 1949 | 16 | 18·8 |
| Guthorn, 1951 | 15 | 27·0 |
| Somerville, 1952 | 19 | 27·4 |
| Huggins and Taylor, 1955 | 75 | 8·0 |
| Treves and Holleb, 1955 | 146 | 29·0 |
| Greening and Aichroth, 1965 | 28 | 36·0 |
| Peltokallio and Kalima, 1969 | 42 | 26·0 |

were diagnosed in them in the same stage as in the female cases (Treves *et al.*, 1956). Taking all the stages of the disease into consideration the prognosis for men is more ominous than for women, but when axillary metastases are present the survival time is the same and when distant metastases are found men live longer on the average than women (Moss, 1965).

The age-adjusted survival rates for our material show that the risk of death from mammary carcinoma is greater among the younger than the older patients. Treves and his co-workers (1956) claimed that age is not of significance for the prognosis; however, the course of the disease appears to be more favourable in patients over 70 years of age.

### SUMMARY

The incidence of both male and female breast carcinoma is lower in Finland than in many other countries. The average male annual morbidity rate in 1953–63 was 0·19/100,000. Men accounted for 0·54 per cent of all cases of mammary carcinoma. The material analysed here consisted of all malignant tumours of the male breast diagnosed and studied histologically in Finland in 1935–63.

There were 51 patients in all. Carcinoma was verified in 42 of these cases. Men appeared to develop the disease almost 10 years later than women. The mean age of the patients was high, 66 years. Two patients had carcinoma in both breasts. Judging by this material, heredity, trauma, gynaecomastia or earlier hormonal therapy play no role in the genesis of carcinoma of the male breast. Men came late for treatment because they could not imagine that they could develop mammary carcinoma. The average delay was 12 months, compared with only 4 months for women. The disease had also spread further in men by the time of diagnosis. The percentage of men operated on in a therapeutically favourable phase was only 47, compared with 70 per cent of the women. Both these factors contributed to making the male prognosis distinctly inferior to that for a corresponding female breast cancer material. Of the total material, 26 per cent of the men and 51 per cent of the women survived over 5 years. The relative 5-year survival rate for men was 34 per cent and for women 54 per cent. The Finnish man had a poorer prognosis than the Finnish woman even when the carcinoma of the mammary gland was treated in an early stage. Classical radical mastectomy is regarded as the therapy of choice also in carcinoma of the male breast. Of the patients that it was possible to treat for cure, 50 per cent were alive after 5 years. Younger patients appeared to have a slightly poorer carcinoma prognosis than older ones who often die of other diseases.

This investigation was aided by a grant from the President J. K. Paasikivi's Foundation, Helsinki, Finland.

### REFERENCES

CAMPBELL, J. H. AND CUMMINS, S. D.—(1951) *Cancer, N.Y.*, **14**, 303.
EDELMAN, S.—(1967) *J. Mt. Sinai Hosp.*, **34**, 578.
EL-GAZAYERLI, M. M. AND ABDEL-AZIZ—(1963) *Br. J. Cancer*, **17**, 566.
FINNISH CANCER REGISTER—(1953–56, 1957, 1958, 1959, 1960, 1961, 1962, 1963) 'Cancer Incidence in Finland'. Cancer Society of Finland publication No. I–VIII, Helsinki.
GILBERT, J. B.—(1933) *Surgery Gynec. Obstet.*, **57**, 451.
GREENING, W. P. AND AICHROTH, P. M.—(1965) *Br. J. Cancer*, **19**, 92.
GUTHORN, P. J.—(1951) *Milit. Surg.*, **109**, 110.
HAKAMA, M.—(1964) *Duodecim*, **80**, 348.
HOLLEB, A. I., FREEMAN, H. P. AND FARROW, J. H.—(1968*a*) *N.Y. St. J. Med.*, **68**, 544.
—(1968*b*) *N.Y. St. J. Med.*, **68**, 656.
HUGGINS, C. AND TAYLOR, G. W.—(1955) *Archs Surg.*, **70**, 303.
JÄÄSKELÄINEN, V.—(1951) *Annls. Med. exp. Biol. Fenn.*, **29**, Suppl. 3.
KOMURDJAEV, H. A. AND ROGOZNAYA, A. V.—(1964) *Vop. Onkol.*, **1**, 87.
LACASSAGNE, A.—(1932) *C.r. hebd. Séanc. Acad. Sci., Paris*, **195**, 630.
MCCLURE, J. A. AND HIGGINS, C. C.—(1951) *J. Am. med. Ass.*, **146**, 7.
MOSS, N. H.—(1965) *In* 'Progress in Clinical Cancer.' New York (Grune J. Stratton Inc.) Vol. 1, p. 515.
PAYSON, B. A. AND ROSH, R.—(1949) *Radiology*, **52**, 220.
PELTOKALLIO, P., KALIMA, T. AND FRILANDER, M.—(1969) *Acta chir. scand.*, in press.
RISSANEN, P. M.—(1968) *Radiologia clin.*, **37**, 129.
RUBIN, P.—(1967) *J. Am. med. Ass.*, **201**, 124.
SOMERVILLE, P.—(1952) *Br. J: Surg.*, **39**, 296.
TREVES, N. AND HOLLEB, A. I.—(1955) *Cancer, N.Y.*, **8**, 1239.
TREVES, N., ROBBINS, G. F. AND AMORSOSO, W. L.—(1956) *Archs Surg.*, **73**, 319.